# Stress, coping profiles and anxiety among French final-year medical students in a high-stakes national ranking examination: a cross-sectional study

**DOI:** 10.1136/bmjopen-2026-125171

**Published:** 2026-09-08

**Authors:** Amandine Mathé, François Tison, François Sztark, Emmanuel Mellet, Alexandre Ouattara, Didier Gruson, Claire Roubaud-Baudron, Igor Sibon, Damien Roux, Patrick Dehail, Pierre Merville, Etienne Riviere

**Affiliations:** 1 Palliative Care, CHU de Bordeaux, Bordeaux, Nouvelle-Aquitaine, France; 2 Service de Neurologie des Maladies Neurodégénératives, IMNc, CHU Bordeaux, Bordeaux, France; 3 CNRS, IMN, UMR 5293, University of Bordeaux, Bordeaux, France; 4 Institut de Médecine Intégrative et Complémentaire (IMIC), Hôpital Xavier Arnozan, CHU de Bordeaux & Université de Bordeaux, Pessac, France; 5 Service d'Anesthésie-Réanimation I, CHU de Bordeaux, Bordeaux, France; 6 Department of Cardiovascular Anesthesia and Critical Care, CHU de Bordeaux, Bordeaux, France; 7 Biologie des Maladies Cardiovasculaires, U1034, Université de Bordeaux & INSERM, Pessac, France; 8 Medical Intensive Care Unit, CHU de Bordeaux, Bordeaux, France; 9 Centre de Recherche Cardio-Thoracique de Bordeaux, INSERM UMR 1045, Université de Bordeaux, Bordeaux, France; 10 Pôle de Gérontologie Clinique, CHU de Bordeaux, Bordeaux, France; 11 Univ. Bordeaux, INSERM UMR 1312, Bordeaux Institute of Oncology, Université de Bordeaux, Bordeaux, France; 12 Stroke Unit, Department of Neurology, Pellegrin Hospital, University Hospital Centre of Bordeaux, Bordeaux, France; 13 University of Bordeaux, CNRS, EPHE, INCIA, UMR 5287, University of Bordeaux, Bordeaux, France; 14 Faculté de Santé, US UPPERS, Unité de Service Pour La Pédagogie, L'Enseignement Et La Recherche, Université Paris Cité, Paris, France; 15 Hôpital Louis Mourier, DMU ESPRIT, Service de Médecine Intensive Réanimation, AP-HP, Colombes, France; 16 Physical and Rehabilitation Medicine Department, University Hospital of Bordeaux, Bordeaux, France; 17 University of Bordeaux, INSERM, BPH, U1219, ACTIVE Team, University of Bordeaux, Bordeaux, France; 18 Department of Nephrology, Transplantation, Dialysis and Apheresis, Bordeaux University Hospital, Bordeaux, France; 19 Centre National de la Recherche Scientifique - Unité Mixte de recherche (CNRS-UMR) 5164 ImmunoConcEpT, Bordeaux University, Bordeaux, France; 20 Dean of the Faculty of Medicine, College of Health Science, Bordeaux University, Bordeaux, France; 21 Internal Medicine and Infectious Diseases Unit, Haut-Leveque Hospital, University Hospital Centre of Bordeaux, Pessac, France; 22 Faculty of Medicine, Bordeaux University, Bordeaux, France

**Keywords:** MEDICAL EDUCATION & TRAINING, MENTAL HEALTH, Mindfulness, Stress, Psychological, Self Care

## Abstract

**Objectives:**

To describe stress, coping behaviours, anxiety, psychological support and benzodiazepine use among French final-year medical students sitting a high-stakes national ranking examination and to explore how these patterns relate to exam ranking when interpreted through transactional stress and coping and ‘coping reservoir’ models.

**Design:**

Cross-sectional questionnaire survey.

**Setting:**

Single university medical faculty in France; final-year students sitting the national *Épreuves Dématérialisées Nationales* (EDN) and ‘national Objective Structured Clinical Examination (nOSCE)’ clinical examination.

**Participants:**

All 387 sixth-year students present for the 2025 nOSCE were invited; 192 (50%) completed the questionnaire (69% women; median age 25 years). Inclusion: registered for the national exam and attending nOSCE; exclusion: non-attendance or incomplete questionnaire.

**Interventions:**

None.

**Primary and secondary outcome measures:**

Primary outcomes were self-rated stress for EDN and nOSCE and *State-Trait Anxiety Inventory-Y* (STAI-Y) state and trait anxiety scores. Secondary outcomes included stress management behaviours, psychological/psychiatric follow-up, benzodiazepine and other psychotropic use and final national ranking.

**Results:**

92% of respondents reported that stress impacted their daily life, particularly attention (83%), sleep (79%) and learning capacity (64%). Overall, 32% had consulted a psychologist/psychiatrist and 15% used benzodiazepines. Most reported regular sport (86%; ≥2 sports 69%) and breathing exercises (61%). Median nOSCE stress was 8/10; median STAI-Y state and trait scores were 45 and 46. Comparing very low versus high/very high trait-anxiety subgroups, ranking did not differ significantly, but low-anxiety students reported more sport (p=0.04), less benzodiazepine use (1/24 vs 10/37; p=0.003) and far less psychological follow-up (1/24 vs 19/46; p<0.0001). Self-reported coping strategies were not associated with better ranking.

**Conclusions:**

Final-year medical students preparing for a high-stakes national ranking examination report pervasive stress, substantial functional impairment and frequent professional and pharmacological coping, with only limited linkage between anxiety and performance. Within transactional and coping reservoir frameworks, these results highlight the need for reflection on structural assessment practices and for longitudinal institutional strategies to protect learner well-being.

**STRENGTHS AND LIMITATIONS OF THIS STUDY**

- Single-centre cross-sectional survey of an entire final-year cohort at a defined high-stakes exam time point, allowing detailed characterisation of stress, coping and anxiety in context.
- Use of a structured, piloted 40-item questionnaire and the validated French *State-Trait Anxiety Inventory-Y* (STAI-Y) state/trait inventory to ensure content and construct validity of key measures.
- Precise linkage of anonymous survey data with objective national ranking via exam registration numbers, while preserving confidentiality and minimising social desirability bias.
- Moderate response rate (50%) and better median rank among responders introduce potential selection bias and limit external validity beyond this faculty.
- Cross-sectional, self-report design precludes causal inference and detailed assessment of frequency, intensity and quality of individual coping strategies.

## Introduction

 Access to postgraduate medical training in France is conditioned by a high-stakes national ranking examination (EN) taken in the final sixth year of medical school. This exam called EN (*Épreuves Nationales,* EN) combines a remote knowledge-based test (*Épreuves Dématérialisées Nationales*, EDN) covering the whole curriculum and a national Objective Structured Clinical Examination (nOSCE).^1^ The EN resulted from a recent reform of medical education first implemented in the 2023–2024 academic year. The national overall ranking arising from these two components governs both specialty choice and allocation to university hospitals for residency, shaping future careers.^2 3^ All 10 000 French final-year medical students take an identical national examination simultaneously in their home faculty to guarantee the best possible equity. This examination, which is both qualifying and ranking, is, therefore, of paramount importance for medical students.^2 3^

High-stakes examinations are known to be potent sources of stress and anxiety among medical students.^4–6^ Beyond transient discomfort, sustained stress can impair cognitive performance, sleep, physical and mental health and may lead to maladaptive coping behaviours, including problematic substance use or excessive self-medication.^7^ Conversely, some degree of activation may enhance motivation and performance, making the relationship between stress and outcomes complex and non-linear.^8^

In the French context, the advent of the reformed EN has added new sources of uncertainty for students, including reliance on remote assessment technologies for the EDN taking place in October, and national standardised OSCE with 10 stations (5/day) taking place at the end of May of the following calendar year. Yet little empirical evidence is available regarding how French final-year medical students experience and manage this stress, which strategies they adopt, and whether these strategies are associated with their eventual ranking. One team has shown that avoidance coping strategy is though detrimental to both well-being and nOSCE performance.^9^ The same team also shown that some students’ personality traits (‘Big Five’)^10 11^ might benefit either from biofeedback^12^ (extroverted trait) or mindfulness^13^ (agreeableness trait).^14^

In framing this work, we drew on Lazarus and Folkman’s transactional theory of stress and coping,^15^ in which stress results from the appraisal of demands relative to personal and contextual resources, and coping strategies are deployed to manage these demands. Within this perspective, medical students’ responses to a high-stakes ranking examination can be understood as patterns of primary appraisal (threat, challenge), secondary appraisal (perceived resources) and coping (behavioural, cognitive and medicalised). We also mobilised the conceptual ‘coping reservoir’ model of medical student well-being, where personal traits, coping style, social support and institutional context jointly determine whether experiences replenish or drain students’ resilience over time.^16^ In this literature, different ‘profiles’ of distress and coping arise depending on how demands are appraised (eg, threat vs challenge), what resources are available and whether coping is behavioural, cognitive, social or medicalised.^16 17^ In our study, profiles of anxiety and coping are operationalised as combinations of *State-Trait Anxiety Inventory-Y* (STAI-Y) state and trait scores, self-reported coping behaviours (sport, breathing exercises, other strategies) and use of professional and pharmacological coping, examined in relation to national ranking within a performance-oriented system.

Using a validated instrument (STAI-Y)^18–20^ and numerical rating scales, we aimed to explore how French final-year medical students prepared for the newly implemented national ranking examination (EN), which stress management strategies they employed and how these related to their anxiety levels and ultimate national ranking outcomes. To our knowledge, no prior study has examined how French final-year medical students experience and manage stress in the context of the newly reformed EN that combines EDN and nOSCE, nor how stress-coping profiles relate to national ranking when interpreted through contemporary stress and well-being models. This study, therefore, addresses a gap between descriptive reports of exam anxiety and conceptual models of stress and coping in health profession education. Guided by Lazarus and Folkman’s transactional theory and the coping reservoir model, we formulated three exploratory hypotheses: (1) students engaging in structured, active coping behaviours (regular physical activity, breathing exercises) would report lower trait anxiety and less reliance on medicalised coping (benzodiazepines and other psychotropics) than those without such behaviours; (2) higher levels of trait anxiety would be associated with greater functional impact of stress and more frequent psychological follow-up but only modestly with final exam ranking and (3) distinct stress-coping profiles (combinations of anxiety, coping behaviours and medicalised coping) would emerge across the ranking spectrum, illustrating different ways students maintain performance within a performance-oriented system.

## Methods

### Study design and setting

A cross-sectional observational study design was chosen for this investigation due to its efficiency in capturing a snapshot of the prevalence and characteristics of stress, anxiety and coping behaviours within a specific population (final-year medical students) at a critical time point (immediately post-nOSCE). This design is well-suited for initial explorations of phenomena within large populations and allowed us to rapidly assess the immediate impact of the newly implemented national examination system.^21^ While precluding causal inferences, it provides valuable descriptive data and identifies associations that can inform future longitudinal or interventional research. This study was conducted among final-year medical students at a French university (sixth year, academic year 2024–2025) who sat the EN in 2025. In the year of this survey, 9525 students registered in France but only 9455 took at least one EDN exam in October 2024. If students obtained a note <14/20 for the essential medical items, they underwent the second retake session: only 9096 students were finally invited to take the nOSCE exam. Finally, 8953 ultimately qualified for the national pairing process to select a specialty and a university for residency after obtaining a minimal overall note of 10/20. The survey was administered immediately after completion of the second day of the nOSCE session in the exit room for the students in our university, that is, the 387 eligible students enrolled at our participating university who were present and took the nOSCE exam in 2025. These 387 students constituted the target population for our single-centre study, representing a fraction of the total national cohort. As this was an exploratory survey of all eligible students at the faculty, no formal a priori power calculation was performed; instead, the sample size was determined by the size of the final-year cohort present at the nOSCE. Within our conceptual framework, this ‘snapshot’ was intended to capture how appraisals, coping behaviours and stress responses co-occur at the peak of a high-stakes examination, as suggested by transactional stress models.

### Participants

All students from the University of Bordeaux present at the nOSCE sessions and enrolled in 6th year were invited to participate. Paper questionnaires were distributed to all students immediately after they had completed the second day of the nOSCE session. This occurred in the designated exit room, a quiet space just outside the examination area. Students were informed about the study’s purpose, reassured of its voluntary and anonymous nature, and invited to complete the questionnaire before leaving the premises. Completed questionnaires were placed in a sealed box by the students themselves to ensure confidentiality. Completion was not individually monitored or timed; students completed the survey independently in the exit room, and staff were present only to distribute and collect forms and to close the room at the end of the session.

### Measures and validity

The study utilised a 40-item structured questionnaire (online supplemental appendix 1) developed by a multidisciplinary team of medical educators and psychiatrists to ensure content validity regarding the French national ranking reform. The instrument was internally piloted with five senior medical students and two faculty members to confirm face validity, logical flow and comprehensibility, but no formal reliability indices (eg, Cronbach’s alpha) were computed; thus, the 40-item questionnaire should be considered a structured data-collection tool rather than a fully validated psychometric scale, and this is acknowledged as a limitation. The final version required approximately 10 min to complete, a duration chosen to minimise respondent fatigue in the post-examination context. The questionnaire comprised three primary domains:

#### Contextual and behavioural data (Q1–9, Q11)

We collected demographics (age and sex), employment status and physical activity patterns. Participation in elective university-led wellness modules was recorded, including an online faculty programme specifically focused on stress reduction for OSCE preparation (‘Stress ECOS’) as well as separate options in medical hypnosis and narrative medicine. Personal coping strategies (eg, breathing exercises, yoga, meditation) were also recorded.

Recourse to professional psychological support and anxiolytic medication (benzodiazepines) during the exam year was also assessed.

#### Functional impact and acute stress (Q10, Q12–17)

Question 10 evaluated the functional impact of stress across four domains: attention, sleep, learning capacity and eating behaviours. Acute perceived stress for the EDN and nOSCE was measured using a 0–10 numerical rating scale, a validated tool for rapid assessment of subjective intensity. To capture the temporal distribution of coping, strategies were specifically tracked in the hours leading up to the nOSCE (Q14), between clinical stations (Q15) and between examination days (Q16).

#### Standardised anxiety assessment: STAI-Y (Q21–40)

To ensure high construct validity, anxiety was measured using the French version of the STAI-Y.^20 22^ This gold-standard psychometric tool comprises two 20-item subscales: the ‘state’ subscale (assessed immediately post-nOSCE) and the ‘trait’ subscale (habitual anxiety). Scores were categorised using established cut-offs: very low (<36), low (36–45), moderate (46–55), high (56–65) and very high (>65). The inventory is recognised for its high internal consistency (Cronbach’s alpha >0.89) and reliability in high-stakes academic settings.^20 22^ In line with transactional stress theory, we considered STAI-Y state scores as reflecting acute stress responses to the examination context, and STAI-Y trait scores as markers of more enduring anxiety dispositions and coping reservoir characteristics.

### Performance at the final national ranking

For each respondent, the final national rank after combination of EDN and nOSCE was extracted (or reported) and considered as a continuous outcome. For descriptive purposes, ranks were also discussed in terms of coarse bands (eg, <1500, <4500, >7000). Among the 9096 participating French students, ranks were obtained and comparisons were made using the inscription number of the students to maintain anonymity. To allow for the matching of survey responses with objective national ranking data while preserving full anonymity, students provided their unique national exam registration number (question 3). As per the protocol approved by our Institutional Review Board (Number UFR-ScMed-UB-2025-01), this identifier was used by an independent administrative officer to link the datasets. Researchers received only a fully deidentified database for analysis, minimising social desirability bias, particularly regarding sensitive disclosures such as medication use or psychological follow-up.

### Statistical analysis

Descriptive statistics summarised demographic characteristics, stress management behaviours, psychological support and medication use, perceived impact of stress, exam-specific stress ratings and STAI-Y scores. Continuous variables are reported as medians with IQRs and minima/maxima; categorical variables as counts and percentages. Between-group comparisons of continuous outcomes (eg, STAI-Y scores, numerical stress ratings, national ranks) were performed using Student’s t-test or Mann-Whitney U test according to distributional assumptions (with ranks treated as non-Gaussian). Categorical variables (eg, presence of psychological follow-up, benzodiazepine use, participation in stress management modules) were compared using Fisher’s exact test. A p value of ≤0.05 was considered statistically significant. Correlation between STAI-Y state and trait scores was examined using linear regression, with the coefficient of determination (R²) summarising shared variance. All analyses were performed using GraphPad Prism V.10 (GraphPad Software, La Jolla, California).

## Results

Descriptive statistics for the overall cohort, disaggregated by gender, are presented in table 1.

**Table 1:** Participant characteristics, stress management behaviours and anxiety levels

Characteristic	Overall cohort (N=192)	Women (n=132, 69%)	Men (n=60, 31%)	P value (women vs men)
Demographics				
Age, median (IQR)	25 (24–25)	25 (24–25)	25 (24–25)	0.21
Concurrent regular paid job, n (%)	28 (15%)	18 (14%)	10 (17%)	0.66
Physical activity				
At least one regular sport, n (%)	165 (86%)	114 (86%)	51 (85%)	0.82
At least two regular sports, n (%)	132 (69%)	86 (65%)	40 (68%)	0.87
No physical activity, n (%)	27 (14%)	20 (15%)	7 (12%)	0.66
Stress management training				
Optional online ‘stress OSCE’ faculty programme, n (%)	32 (17%)	28 (21%)	4 (7%)	0.01*
Optional medical hypnosis faculty module, n (%)	20 (10%)	13 (10%)	7 (12%)	0.8
Narrative medicine faculty module, n (%)	8 (4%)	6 (4.5%)	2 (3%)	0.99
Other trainings, n (%)	14 (7%)	9 (7%)	5 (8%)	0.76
Self-reported stress management strategies				
Cardiac coherence/abdominal breathing, n (%)	117 (61%)	88 (67%)	30 (50%)	0.04*
Yoga (as specific strategy), n (%)	5 (3%)	5 (4%)	0 (0%)	0.05*
Mindfulness meditation, n (%)	2 (1%)	1 (1%)	1 (2%)	0.53
Self-hypnosis, n (%)	2 (1%)	2 (2%)	0 (0%)	0.99
Other informal strategies, n (%)	15 (8%)	11 (8%)	4 (7%)	0.76
Used at least two distinct strategies, n (%)	62 (32%)	42 (32%)	20 (33%)	0.8
Reported no specific strategy, n (%)	50 (26%)	39 (29%)	11 (18%)	0.06
Neither sport nor specific strategy, n (%)	6 (3%)	4 (3%)	2 (3%)	0.9
Psychological follow-up and medication				
Consulted psychologist/psychiatrist, n (%)	61 (32%)	50 (38%)	11 (18%)	0.007*
Used benzodiazepines (previous year), n (%)	29 (15%)	22 (17%)	7 (12%)	0.51
Used other psychotropic medications*, n (%)	15 (8%)	13 (10%)	2 (3%)	0.15
Perceived impact of stress on daily life				
No impact reported, n (%)	16 (8%)	6 (4.5%)	10 (17%)	0.009*
Impact on attention/concentration, n (%)	159 (83%)	121 (92%)	38 (63%)	0.0001*
Impact on sleep quality, n (%)	151 (79%)	111 (84%)	40 (68%)	0.008*
Impact on learning capacity, n (%)	123 (64%)	95 (72%)	28 (47%)	0.001*
Impact on eating patterns, n (%)	94 (49%)	73 (55%)	21 (35%)	0.01*
Reported at least two distinct impacts, n (%)	149 (78%)	116 (88%)	33 (55%)	0.0001*
Stress and anxiety levels				
Self-rated stress EDN (0–10), Median (IQR)	7 (5–8)	7 (6–8)	6.5 (5–8)	0.1
Self-rated stress nOSCE (0–10), Median (IQR)	8 (6–9)	8 (6–8)	6.5 (5–8)	0.002*
STAI-Y State (N=176, W=125, M=51), median (IQR)^†^	45 (38–56)	48 (39–57)	40 (34–48)	0.0008*
STAI-Y Trait (N=176, W=125, M=51), median (IQR)^†^	46 (39–56)	49 (42.5–57)	39 (34–46)	<0.0001*

* Includes antidepressants (n=6), hydroxyzine (n=4), neuroleptic (n=1), cannabidiol (n=1), zolpidem (n=1), beta-blocker (n=1).

† n=176 for STAI-Y measures due to incomplete responses from some participants. P value is considered significant if alpha=0.05.

EDN, Épreuves Dématérialisées Nationales; nOSCE, national Objective Structured Clinical Examination; STAI-Y, State-Trait Anxiety Inventory-Y.

### Participant characteristics

Among the 387 eligible students, 192 completed the questionnaire (response rate 50%). Women represented 69% of the sample and men 31%. Median age was 25 years (IQR 24–25; range 23–35). Twenty-eight students (15%) reported having a regular paid job in parallel with their final-year studies with similar proportions across genders (14% women vs 17% men, p=0.66). The median national rank for the 192 respondents was 3360 (IQR 1514–5658), which was significantly better than the median rank of 4880 (IQR 2637–6888) for the 195 non-responding eligible students at our university (p<0.0001, Mann-Whitney U test, assuming non-Gaussian distribution appropriate for ranks).

### Physical activity and stress management training

Regular physical activity was common, with 86% (n=165) engaging in at least one regular sport and 69% (n=132) practising at least two sports. No significant gender differences were observed in overall sport participation or in the proportion reporting no physical activity (14% overall; 15% women vs 12% men, p=0.66).

Exposure to formal stress management or reflective practice training was as follows (representative of the whole population): 17% (n=32) attended the online ‘stress OSCE’ faculty programme, with a significantly higher proportion of women participating compared with men (21% vs 7%, p=0.01). Participation in other optional modules, such as medical hypnosis (11% overall), narrative medicine (4% overall) or other trainings (7% overall), did not show significant gender differences.

### Stress management strategies during the exam year preceding EDN then nOSCE

Regarding personal strategies to manage stress, 61% (n=117) reported practising cardiac coherence and/or abdominal breathing exercises, with a significant difference between women and men (p=0.04). Specific use of yoga as a stress management strategy was reported by a small number of students (3% overall), exclusively by women (5 women vs 0 men, p=0.05). The prevalence of mindfulness meditation (1%), self-hypnosis (1%) and other informal strategies (8%) was low, with no significant gender disparities. Other strategies included video or board games, increased social interaction, relying on a pet (often a cat), cannabis, Bach flowers (naturopathy), regular prayer or even using selective serotonin reuptake inhibitors in self-medication. Overall, 32% (n=62) used at least two distinct strategies, while 26% (n=50) reported no specific strategy, and only 3% (n=6) reported neither regular sport nor any stress management technique; none of these showed significant gender differences.

### Psychological follow-up and medication

Recourse to professional psychological support was significantly higher among women, with 38% (n=50) of women reporting having been followed by a psychologist or psychiatrist, compared with 18% (n=11) of men (p=0.007). Benzodiazepine use during the preceding year was reported by 15% (n=29) of students, with no significant gender difference (22 women vs 7 men, p=0.51). Fifteen students felt free to specify the use of additional therapeutic interventions, although this information was not explicitly requested, and these additional treatments reported included antidepressants (n=6), hydroxyzine (n=4), neuroleptic (n=1), cannabidiol (n=1), zolpidem (n=1), beta-blocker (n=1). These data highlight a non-trivial recourse to pharmacological strategies to manage exam-related anxiety.

### Perceived impact of stress on daily life

While only 8% (n=16) declared no impact of stress on daily life, men were significantly more likely to report no impact compared with women (17% vs 4.5%, p=0.009). The perceived impact of stress on daily life was pervasive and significantly higher among women across multiple domains. Women reported a higher impact on attention and concentration (92% vs 63% in men, p=0.0001), sleep quality (84% vs 68% in men, p=0.008), learning capacity (72% vs 47% in men, p=0.001) and eating patterns (55% vs 35% in men, p=0.01). Overall, 78% (n=149) reported at least two distinct impacts of stress on quality of life, with women significantly more likely to report multiple impacts (88% vs 55% in men, p=0.0001).

### Exam-specific stress: EDN versus nOSCE

**Figure 1:**
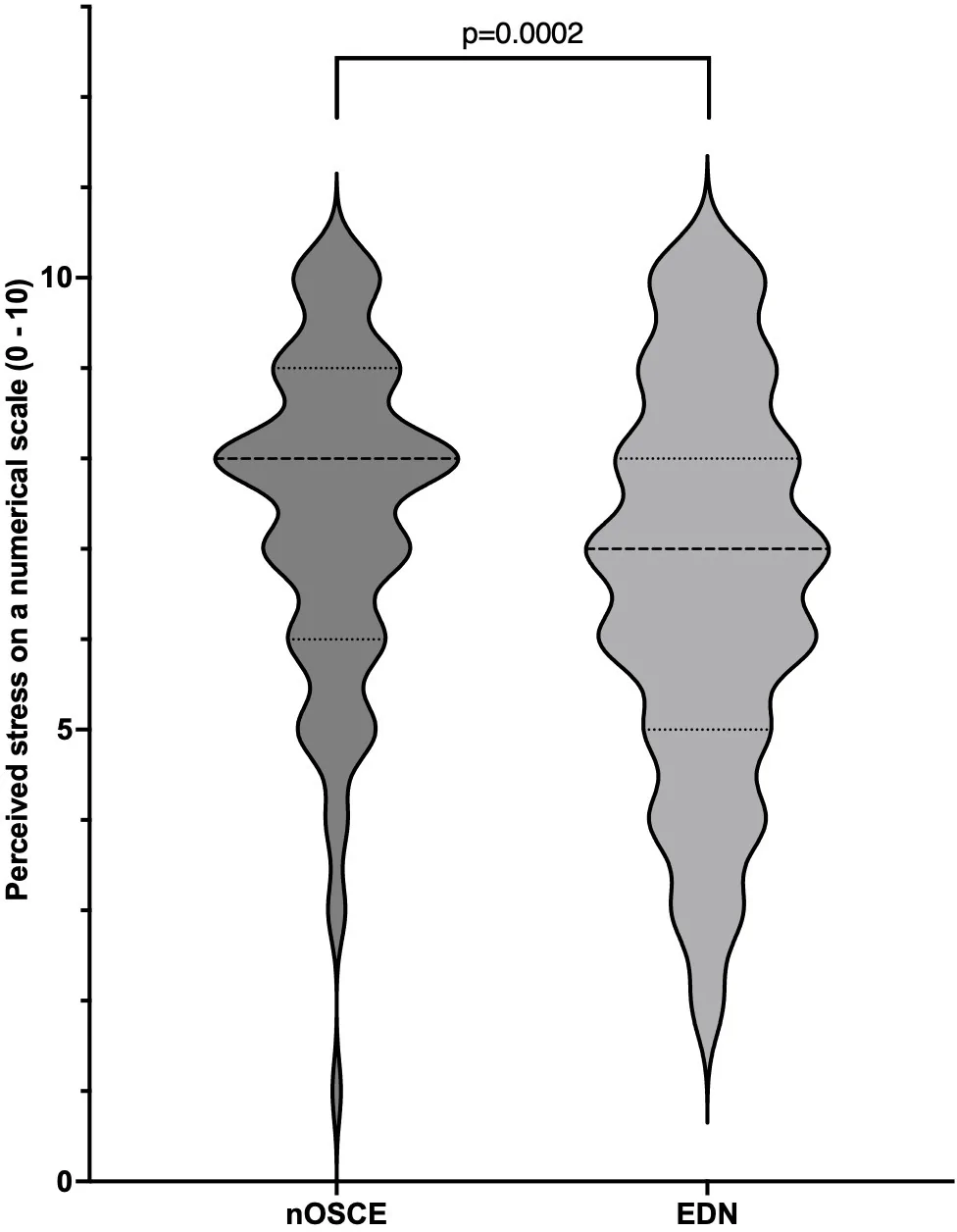
Perceived stress level on a numerical scale between the competency assessment exam (nOSCE) and the knowledge exam (EDN) of medical students for the national ranking exam in 2025, distribution as a violin plot. nOSCE-related stress was rated slightly higher than EDN-related stress on a 0–10 scale, with most students reporting high stress for both components. The width of the violin represents kernel density estimation. The central dashed line indicates the median, and the upper and lower dashed lines represent the first and third quartiles. Apparent extension beyond the scale limits (0–10) reflects density estimation and does not indicate observed values outside the scale. EDN, *Épreuves Dématérialisées Nationales;* nOSCE, national Objective Structured Clinical Examination.

Median stress level was higher at 8/10 (IQR 6–9) for the nOSCE compared with 7/10 (IQR 5–8) for the knowledge exam (EDN, p=0.0002, figure 1). Self-rated stress for the nOSCE (median 8/10) was higher in women compared with men (p=0.002, table 1).

### Anxiety levels: STAI-Y state and trait

**Figure 2:**
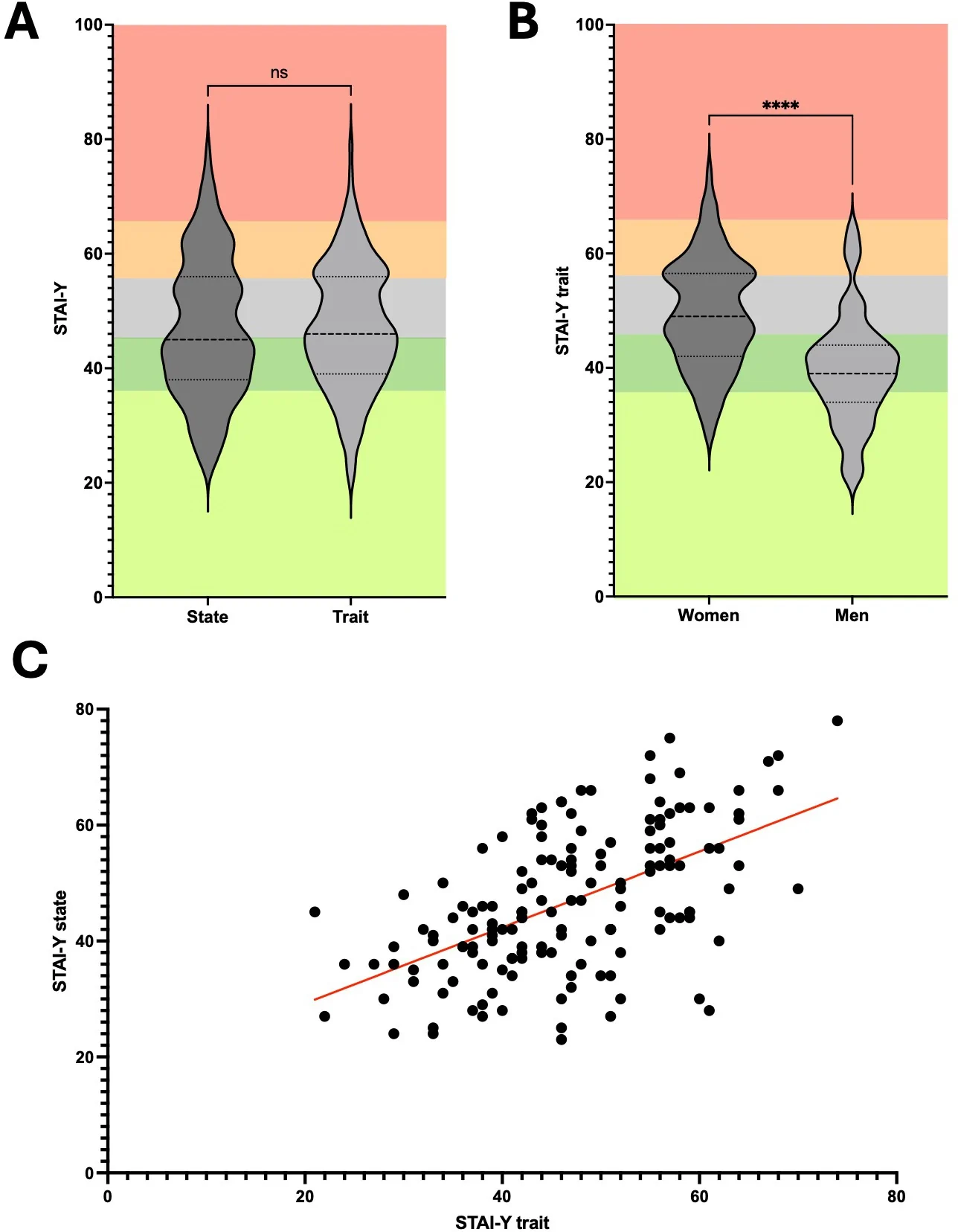
Results of the STAI-Y in the global cohort: STAI-Y state and trait scores were generally in the low-to-moderate range, with women showing higher median trait anxiety than men and only a moderate correlation between state and trait anxiety. (**A**) Results of the state and trait components of the STAI-Y; (**B**) comparison of the trait profiles between women and men; (**C**) correlation between state and trait of the STAI-Y. Distribution as a violin plot: the width of the violin represents kernel density estimation. The central dashed line indicates the median, and the upper and lower dashed lines represent the first and third quartiles. Apparent extension beyond the scale limits (0–10) reflects density estimation and does not indicate observed values outside the scale. Colours: light green, STAI-Y<36 (very low anxiety); green, STAI-Y 36–45 (low anxiety); grey, STAI-Y 46–55 (moderate anxiety); orange, STAI-Y 56–65 (high anxiety) and red, STAI-Y>66 (very high anxiety). STAI-Y, *State-Trait Anxiety Inventory-Y.*

A total of 176 students (92%) provided complete answers to the two STAI-Y subscales: STAI-Y data were missing for 7/132 women and 9/60 men. For this group, median STAI-Y state score (immediately after nOSCE) was 45 (IQR 38–56; range 23–78) and median STAI-Y trait score (habitual anxiety) was 46 (IQR 39–56; range 12–79, figure 2A). The median STAI-Y trait was significantly higher in women compared with men (49 *vs* 39, p<0.0001, figure 2B, table 1). According to established categories, 24 (13.6%) had very low anxiety, 56 (32%) had low anxiety, 50 (28.4%) had moderate anxiety, 39 (22%) had high anxiety and 7 (4%) had very high anxiety. There was not only a statistically significant but only a moderate correlation between state and trait scores, with r²=0.32 (figure 2C), suggesting that while students’ usual anxiety predisposition partly explained situational OSCE-related anxiety, the immediate exam context might have contributed to elevated state anxiety.

### Trait anxiety and final national ranking

**Figure 3:**
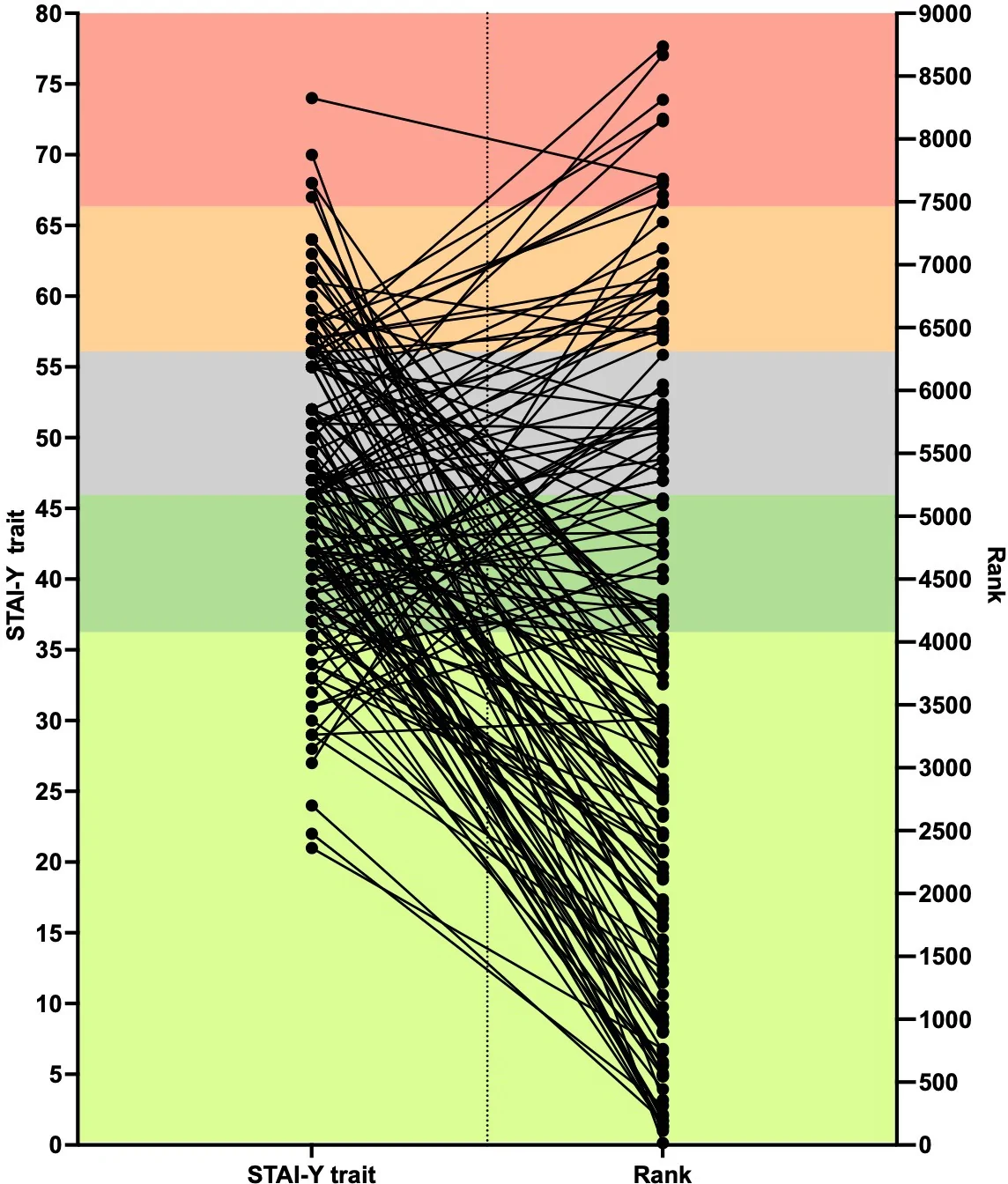
Pair match between the STAI-Y trait score and the final rank obtained after the national ranking exam: across the ranking spectrum, many students with low-moderate trait anxiety achieved good rankings, and extreme anxiety profiles (very low vs very high) were distributed across both high and low ranks, illustrating weak linkage between trait anxiety and performance. Colours: light green, STAI-Y<36 (very low anxiety); green, STAI-Y 36–45 (low anxiety); grey, STAI-Y 46–55 (moderate anxiety); orange, STAI-Y 56–65 (high anxiety) and red, STAI-Y>66 (very high anxiety). STAI-Y, *State-Trait Anxiety Inventory-Y.*

To explore the relationship between habitual anxiety and performance, students were classified by trait STAI-Y level and final ranking after the EN (out of 9096 students in the overall population of 6th-year French students in 2025). Overall, most students with very low, low or moderate trait anxiety achieved a better ranking (<1500 or <4500, figure 3). We see that most students with very low to moderate trait anxiety obtained a rank <4500 and that the median rank among responders was 3360 (IQR 1514–5658), lower than the overall population of the 387 students with a median rank of 3433 (Q1-3:1441-5854).

**Figure 4:**
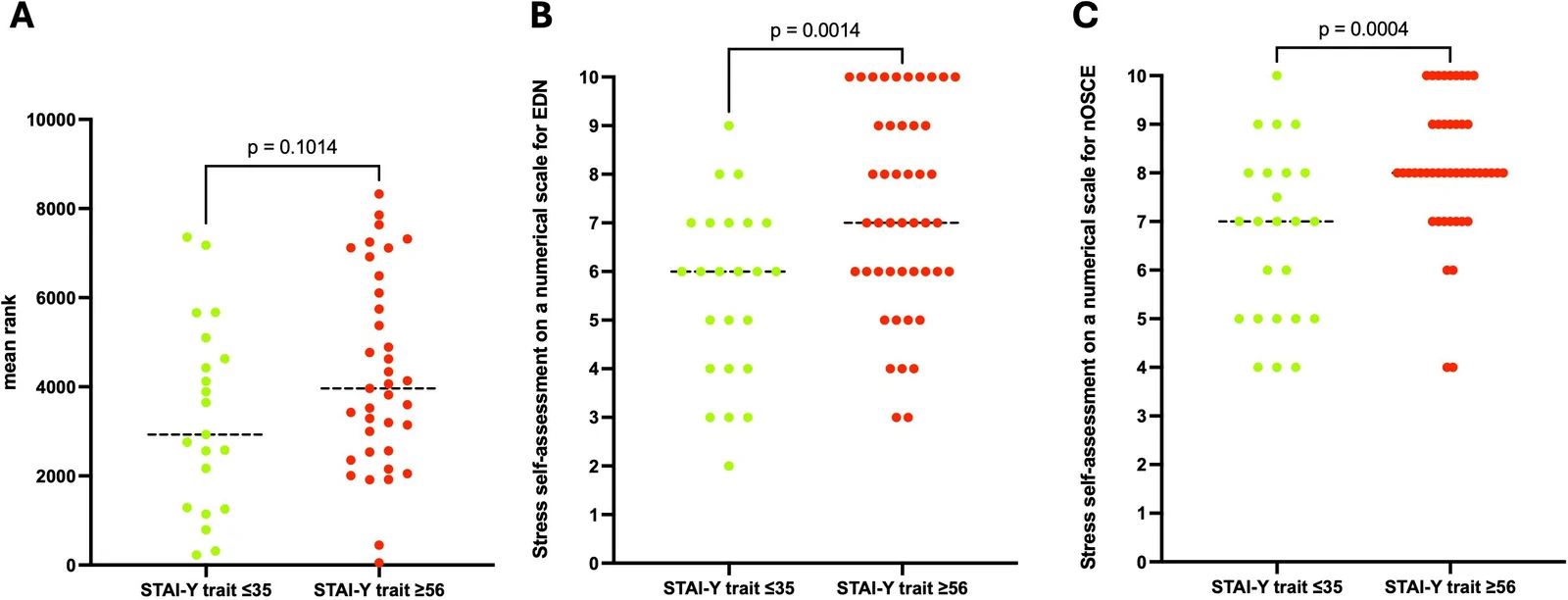
Subgroup analyses of students with extreme STAI-Y trait results. (**A**) Mean rank after the national exam; (**B**) stress self-assessment on a numerical scale for the knowledge exam (EDN); (**C**) stress self-assessment on a numerical scale for the competency exam (nOSCE). Student’s t test, p<0.05 defined significance. EDN, *Épreuves Dématérialisées Nationales;* nOSCE, national Objective Structured Clinical Examination; STAI-Y, *State-Trait Anxiety Inventory-Y.*

We could identify two ‘extreme’ subgroups: a group with very low trait anxiety (<36, n=24) and a group with high/very high (>55, n=46) trait anxiety. These groups form contrasting stress-coping profiles: students with very low trait anxiety but diverse ranking outcomes and students with high/very high trait anxiety who also span the performance spectrum. To compare ranks in the final exam, we had the data for 20/24 students from the very anxiety group, and 37/46 for the high/very high group: there was no significant difference in final national rank (p=0.1, figure 4A). Self-rated stress at EDN (figure 4B) and nOSCE (figure 4C) remained high in both groups, with significant differences in patterns but consistently elevated scores. Students in the very low trait-anxiety group were significantly more likely to practise at least one sport (p=0.04), they were less likely to have used benzodiazepines in the previous year (1/24; p=0.003), and only one had received psychological follow-up versus 19 in the high/very high trait-anxiety group (p<0.0001), although 27 in the latter group had not sought such follow-up. Of note, all the 24 students with high/very high trait anxiety had at least one repercussion of stress on their daily life either on sleep, attention, learning or food intakes compared with 10 out of 24 students with very low trait-anxiety (p<0.0001). Seven students (15%) of the high/very high trait-anxiety group also had supplemental job versus 7 (29%) in the low trait-anxiety group (p=0.2). Taken together, these profiles illustrate that similar ranking outcomes can arise from very different configurations of appraisal, coping resources and stress responses, which is consistent with transactional and coping reservoir models.

### Stress management strategies and performance

**Figure 5:**
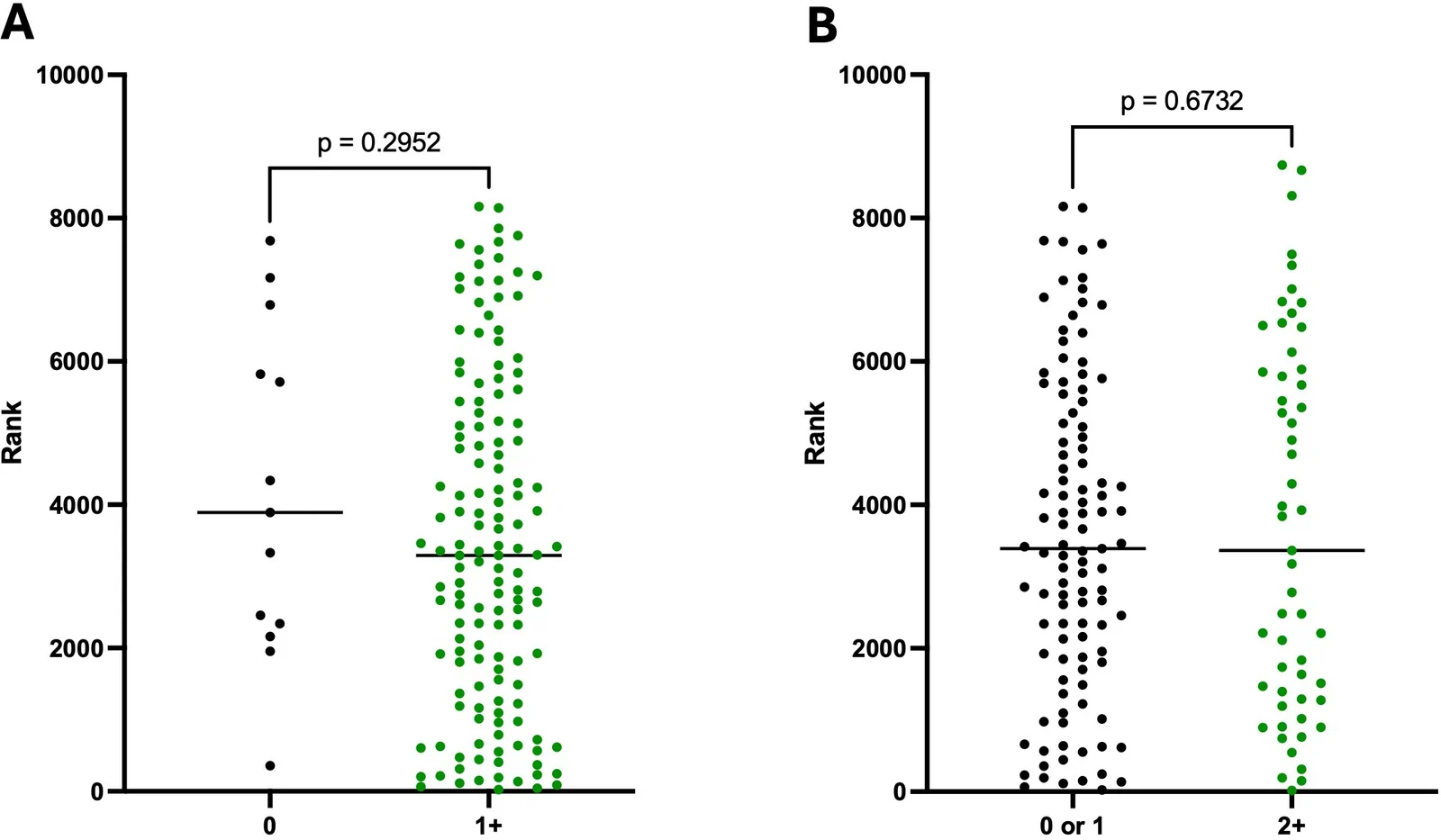
Comparison of the final rank obtained after the national exam according to the number of strategies used to cope with stress and anxiety. (**A**) No strategy versus one or more; (**B**) none or one versus two or more strategies. Student’s t test, p<0.05 defined significance.

Analyses across the entire cohort examined whether adopting specific stress management strategies was associated with better ranking at the nOSCE. No significant differences in rank were observed between students who used at least one stress management strategy and those who used none (figure 5A) or between students who used a single strategy and those who used multiple strategies (figure 5B). Similarly, no beneficial or detrimental effect on ranking was demonstrable for individual approaches (eg, cardiac coherence, yoga, meditation), although the study was not powered to detect small differences or to evaluate strategy quality or adherence in detail.

## Discussion

This study examined stress, coping behaviours, anxiety and national ranking among French final-year medical students sitting a newly implemented high-stakes national examination (EN), and interpreted the observed profiles of distress and coping through transactional and coping reservoir frameworks. Within a transactional stress lens, our findings can be understood as patterns of appraisal and coping: students face high external demands (the EN), appraise these demands as threat or challenge, draw on available resources (sport, breathing exercises, psychological support) and adopt coping responses that are behavioural, cognitive, social or medicalised. The coping reservoir model further suggests that repeated exposure to such demands and coping choices either depletes or replenishes students’ resilience over time, which helps explain how some students maintain performance despite high anxiety and extensive medicalised coping, while others maintain well-being with more moderate performance.

In our cohort, stress was pervasive and intense: students reported high numerical stress scores for both exam components, especially the nOSCE, and a broad impact on attention, sleep, learning and eating, with additional repercussions on social life, mood and somatic health. The majority reported at least two domains of impaired quality of life. Such multidomain impact suggests that exam-related stress extends well beyond the examination period and penetrates daily functioning during the final year, confirming what was previously described and what has been suggested in other academic settings with high-stakes exams.^9 23 24^ Of note, this impact was particularly pronounced among women, who reported significantly higher rates of negative effects on attention, sleep, learning capacity and eating behaviours, suggesting a greater overall burden of stress on their quality of life compared with men. Interpreting these findings through a transactional stress lens highlights how a performance-oriented national ranking examination creates sustained appraisal of threat and loss of control, draining students’ coping reservoirs and leading to multidomain functional impairment even when measured anxiety scores appear only ‘moderate’ on standard inventories.^16^

Second, while overall median STAI-Y scores were in the low-to-moderate range for the cohort, the significantly higher STAI-Y state and trait scores observed in women compared with men indicate a heightened predisposition and acute experience of anxiety in the female student population. Interestingly, despite these objective differences in anxiety and reported impact, self-rated stress intensity for the EDN and nOSCE did not significantly differ by gender, suggesting that while women may experience and report the effects of stress more pervasively, the subjective feeling of acute pressure is broadly shared. State and trait anxiety showed only a modest correlation, reinforcing the idea that situational exam stress can acutely elevate anxiety even in students not habitually anxious, while some highly anxious individuals may cope better than expected during the exam itself. Interestingly, the weak association between trait anxiety and final ranking suggests that anxiety and stress are only modest predictors of academic achievement in this high-stakes context, echoing prior work on OSCE performance and exam anxiety.^4 25^ From a transactional perspective, this may reflect an ‘inverted U’ relationship, where both very low and very high stress can coexist with high performance depending on how demands are appraised and coping resources mobilised, while the coping reservoir model emphasises that performance can be maintained at the cost of progressive depletion of well-being. Our profiles of highly anxious high performers versus minimally anxious low performers illustrate these different trajectories. The median STAI-Y trait score of 46 observed in our sample is consistent with ranges reported in other European medical student cohorts during examination periods, suggesting that while French students are not uniquely more anxious than their international peers, they nonetheless operate at a high baseline of psychological pressure. The discrepancy between ‘moderate’ anxiety scores and ‘substantial’ self-reported impact on daily life suggests that traditional anxiety inventories may not fully capture the cumulative burden of high-stakes competitive ranking on students’ functional quality of life.

Third, students deployed a wide variety of stress management strategies, ranging from structured practices including cardiac coherence, breathing exercises, yoga, meditation or self-hypnosis to more informal methods such as games, social interaction, pets, religious practices or complementary therapies. Over 70% practised sport that has demonstrated significant effect on lowering chronic stress^26 27^ or anxiety,^28 29^ and 60% practised cardiac coherence or abdominal breathing, reflecting its accessibility and the potential perceived efficacy of specific teaching interventions. Of note, it has been shown that regular participation in yoga, mindfulness meditation or walking sessions, for 1 hour two times per week, is associated with lower anxiety and perceived stress.^29 30^ Yet almost a quarter of students reported no specific strategy, and a very small number reported neither physical activity nor deliberate stress management techniques. This vulnerable subgroup may already be engaged in avoidance strategies requiring close institutional monitoring given their negative impact on well-being and proficiency.^9^

Fourth, recourse to professional psychological support and psychotropic medications was frequent as one-third of students reported regular contact with a psychologist or psychiatrist for stress management, and 15% used benzodiazepines during the year, in addition to other psychotropic agents. It has been reported that such use of either psychostimulants like nicotine, caffeine or energy drinks^31 32^ or anxiolytics or antidepressants^33^ is used in up to 40% of medical students, and so in variable cultural contexts across the world.^32–39^ Moreover, our data reveal a significantly higher rate of consultation with a psychologist or psychiatrist among women that could reflect either higher levels of need among women or a greater willingness to seek help. This, combined with the observed high and pervasive impact of stress on daily life for women, strengthens the argument for comprehensive, easily accessible and potentially gender-sensitive psychological support services within medical schools. Overall, these results can be analysed in two ways: (1) the half-empty glass, raising concerns for their future professional practice as physicians or (2) the half-full glass, with a more proactive capacity to seek for help and psychological support, an interesting point to be highlighted for their professional identity formation to ultimately shape well-balanced physicians. In coping reservoir terms, frequent benzodiazepine use and intensive psychological follow-up may be viewed as attempts to stabilise an already depleted reservoir under escalating demands, whereas behavioural strategies such as sport and breathing practices may help replenish resources and reduce reliance on pharmacological coping.^16 40^ Conceptually, our data illustrate how learners can maintain performance while drawing heavily on medicalised coping, raising questions about the trade-offs between short-term success and long-term professional resilience.

However, the relationship between anxiety, coping behaviours and performance appeared complex and weakly deterministic. Neither the presence nor the multiplicity of self-reported stress management strategies was associated with better ranking at the nOSCE. What differed more clearly were coping profiles: very anxious students were more likely to have psychological follow-up and to take benzodiazepines, whereas very low-anxiety students more frequently engaged in sport and rarely used benzodiazepines or specialised care. The most anxious students are also likely to have higher standards and be more self-critical, which can be a source of psychological distress, rumination and ‘internal stress’.^41^ Our data also reveal some gender differences as women were significantly more likely to use yoga as a specific strategy and to participate in the ‘stress OSCE’ faculty programme, and these patterns, alongside higher rates of psychological follow-up, suggest a proactive approach by women to manage their distress, even if these efforts did not translate into a direct academic ranking advantage in this cross-sectional study. Rather than indicating that coping strategies are ineffective, this pattern suggests that in a strongly performance-driven system, such strategies may primarily protect well-being and reduce reliance on medicalised coping (eg, benzodiazepines), without necessarily translating into immediate ranking advantages. Transactional stress models and student coping frameworks similarly emphasise that social support, emotion-focused coping and lifestyle behaviours buffer stress responses and long-term mental health more than they directly enhance short-term examination scores.^42 43^

Taken altogether, these findings suggest that (1) high performance is possible even under intense anxiety, perhaps at the cost of considerable psychological suffering; (2) low subjective anxiety does not guarantee high performance; (3) adaptive lifestyle factors such as regular sport may be associated with lower trait anxiety and reduced reliance on pharmacological coping, although causal directions cannot be inferred; and (4) simply ‘having strategies’ is not sufficient to guarantee a ranking advantage—quality, timing, personal fit and context likely determine their effectiveness for both performance and well-being. Nonetheless, within a coping reservoir perspective, students reporting no specific strategies and minimal physical activity may represent a subgroup at greater risk of emotional exhaustion, avoidance coping or increased reliance on pharmacological strategies, even if this did not manifest as poorer ranking in our cross-sectional data.

From an educational and institutional perspective, these results argue for a multimodal approach to stress and anxiety observed among final-year medical students earlier in their curriculum, and possibly at each major checkpoint.

At the structural level, reflection is needed on the design of high-stakes examinations whose outcomes decisively shape careers. While national ranking procedures are objective and fair, they concentrate pressure within a narrow time window. Ensuring transparent assessment criteria, providing formative nOSCE experiences, and offering multiple exam sessions or remediation pathways may reduce the perceived catastrophic consequences of a single examination.

Integrating mandatory, longitudinal teaching in mental health literacy, self-care and reflective practice throughout the curriculum, rather than as isolated end-of-programme options, could normalise help-seeking and equip students with effective coping tools, an approach that has demonstrated effectiveness in other academic settings.^44–49^ In accordance with a part of our results and what has been shown in the literature regarding mental health of medical students, we believe that institutions should ensure confidential, easily accessible counselling and psychological support independent from assessment structures, along with clear guidance on rational psychotropic use (particularly benzodiazepines) in collaboration with healthcare services. Proactive screening should target at-risk students, including those reporting no coping strategies, no physical activity, marked functional impairment or multiple psychotropic medications (see box 1).

Box 1 **Practical points for educators and policymakers**

- High-stakes national exams produce substantial, multidomain stress in final-year medical students, independent of ultimate performance.
- Many students adopt simple, low-cost strategies (cardiac coherence breathing, sport), yet a significant proportion remain without structured coping approaches.
- Use of benzodiazepines and other psychotropic medications for exam-related stress is non-negligible and warrants targeted guidance and support.
- Anxiety level alone is a poor predictor of ranking; attention should shift from ‘stress tolerance’ to fostering healthy coping and institutional responsibility.
- Integrating structured, longitudinal self-care and mental health curricula, and ensuring confidential access to psychological support, are key priorities in medical education reform.

This study has several strengths. It focuses on a critical transition point in French medical education, immediately after the high-stakes nOSCE, capturing fresh perceptions of stress. It combines subjective ratings (numerical scales, STAI-Y) with objective performance measures like national ranking. It also examines specific coping strategies, professional support and medication use, and explores contrasting extreme profiles.

However, limitations must be acknowledged. The study is single-centre and may not generalise to all French faculties, which might differ in preparation, nOSCE organisation or student support services.^50^ In addition, while the bespoke 40-item questionnaire underwent face-validity piloting, we did not conduct a full psychometric validation including internal consistency analyses, so findings based on these items should be interpreted with this limitation in mind and complemented by the more robust STAI-Y measures. The response rate of 50% opens the possibility of selection bias.^51^ Indeed, a comparison of the national ranks revealed that participating students had a significantly better median national rank than non-participating students (median 3360 vs 4880, p<0.0001). This indicates a performance-related selection bias, where higher ranked students were more likely to participate. This academic bias makes it challenging to definitively interpret the implications for the reported stress and anxiety levels of the entire student population. On one hand, it is possible that students who performed less well (the non-responders) also experienced higher levels of stress, perhaps due to struggles, disappointment or perceived failure relative to their own aspirations. In this scenario, our study would *underestimate* the true prevalence and severity of stress and anxiety in the overall cohort. On the other hand, non-participating students with lower ranks might have had different aspirations, such as aiming for less competitive specialties like family medicine or psychiatry, which inherently require a lower ranking (around half of all students ultimately choose family medicine). If these students achieved their goals with less pressure, their stress and anxiety levels could have been lower than those reported by our higher-achieving participant group, thus potentially leading to an *overestimation* of overall student distress in our findings. Without data on the motivations for non-participation or the personal ranking objectives of the non-responding students, we cannot definitively ascertain the specific direction of this bias on our stress and anxiety metrics. Moreover, all behavioural and clinical data, including medication use, are self-reported and may be influenced by recall or social desirability biases.^52^ We did not collect detailed information on prior academic performance, socioeconomic status or baseline mental health (eg, pre-existing anxiety or depressive disorders), which limits our ability to disentangle the relative contributions of these factors to both anxiety and ranking and introduces potential residual confounding. The cross-sectional design prevents conclusions about causal relationships between strategies, anxiety and performance.^21^ Furthermore, STAI-Y state scores were collected immediately after completion of the nOSCE; they may, therefore, reflect a composite of anticipatory anxiety, perceived performance, relief and fatigue, which could dilute the specificity of ‘during-exam’ stress and should be interpreted accordingly. Measures of frequency, intensity and quality of each strategy were limited, potentially masking differential effectiveness.^52^ Given the descriptive and exploratory nature of this initial study, and the predominantly null findings regarding the association between stress management strategies and performance, extensive multivariate modelling with adjustment for all potential confounders (eg, gender, prior academic performance) was not undertaken as our primary aim was to identify broad patterns and associations, acknowledging that more complex causal inferences would require longitudinal designs and advanced statistical approaches. Finally, the subgroup analyses of extreme profiles, while illuminating, involve small numbers and should be interpreted cautiously: these findings are hypothesis-generating rather than definitive, particularly regarding the absence of a statistically significant difference in ranking between these groups, which might be due to insufficient power to detect a true, small effect

### Future research directions

Building on our findings, future research should pursue longitudinal designs to establish causal relationships between stress, anxiety, coping strategies and long-term well-being and academic outcomes. Qualitative studies are needed to delve deeper into the nuances of stress management, exploring the quality, consistency and personalised aspects of adopted strategies. Furthermore, controlled intervention studies evaluating the effectiveness of specific, evidence-based mental health curricula or support programmes within the French medical education context would provide crucial insights for reform.

## Conclusion

This study investigated the relationship between stress management, anxiety and performance; our hypothesis that specific strategies would be associated with a ranking advantage was not corroborated. Although median anxiety scores remained in the low-to-moderate range, the perceived intensity of stress was high, and its impact on sleep, concentration and learning was nearly universal. Conceptually, the results highlight that high-stakes ranking examinations can produce diverse stress–coping configurations, with similar ranking outcomes arising from very different combinations of anxiety, behavioural coping and medicalised coping, which supports the use of transactional and coping reservoir models to understand learner well-being in performance-oriented systems. Coupled with the observed 15% rate of benzodiazepine use and 32% rate of professional psychological consultation, our findings suggest that high-stakes ranking assessments warrant critical reflection from the perspective of learner well-being and coping capacity, and they provide empirical grounds for institutions to consider structural and curricular approaches that may mitigate documented stress and functional impairment, while recognising that causal and policy inferences require further longitudinal and interventional research.

## Supplementary material

10.1136/bmjopen-2026-125171 online supplemental file 1

## Data Availability

Data are available upon reasonable request.
