# Supplementary material for: Stress, coping profiles and anxiety among French final-year medical students in a high-stakes national ranking examination: a cross-sectional study
Source: BMJ Open. 2026 Sep 8;16(9):e125171. doi: 10.1136/bmjopen-2026-125171 (PMC13560983; doi:10.1136/bmjopen-2026-125171)
Supplement: Supplementary file 1 [file bmjopen-2026-125171.reviewer_comments.pdf]

PEER REVIEW HISTORY

BMJ Open publishes all reviews undertaken for accepted manuscripts. Reviewers are asked to complete a checklist review form and are provided with free text boxes to elaborate on their assessment. These free text comments are reproduced below.

ARTICLE DETAILS

Title (Provisional)

Stress, coping profiles and anxiety among French final-year medical students in a high-stakes national ranking examination: a cross-sectional study

Authors

Mathé, Amandine; Tison, F.; Sztark, François; Mellet, Emmanuel; OUATTARA, Alexandre; Gruson, Didier; Roubaud-Baudron, Claire; Sibon, Igor; Roux, Damien; Dehail, Patrick; Merville, Pierre; Riviere, Etienne

---

VERSION 1 - REVIEW

**Reviewer**	**1**
**Name**	**Tamam, Lut**
**Affiliation**	**Çukurova University Medical School**
**Date**	**29-Jul-2026**
**COI**	**None**

The current study examines stress, coping behaviors, and anxiety among 192 French final-year medical students (50% response rate) preparing for a high-stakes national ranking examination (EN). The study found pervasive stress affecting 92% of students, with significant impacts on attention (83%), sleep (79%), and learning capacity (64%). While 86% engaged in regular sports and 61% practiced breathing exercises, 32% had consulted a psychologist/psychiatrist and 15% used benzodiazepines. Trait anxiety levels did not significantly correlate with final national ranking, and self-reported coping strategies showed no association with better performance. The study is an original one within the scope of the journal. My comments are as follows;

1. The subtitle "a transactional stress perspective" suggests inclusion of theoretical depth in the study which is not the case. It should be removed or revised.

2. Lazarus & Folkman's transactional theory and the "coping reservoir" model are mentioned briefly but not operationalized. They should be integrated into testable hypotheses with clear variables.

3. Hypothesis should be more clear and straightforward with proposed direction of associations.

4. The novelty of the study and the gap it fills out should also be mentioned.

5. The scales are administered "immediately post-nOSCE," the STAI-Y state scores reflect post-exam relief or exhaustion as much as during-exam anxiety. This might compromise validity. It should be mentioned in limitations in discussion.

6. The 40-item instrument used in the study was "developed by a multidisciplinary team" and piloted with only 5 students + 2 faculty. This is insufficient for establishing psychometric properties. Reliability metrics (Cronbach's alpha) should be reported for this instrument.

7. Absence of data on prior academic performance, socioeconomic status, or baseline mental health history that could be crucial for explaining both anxiety and ranking, is an important limitation that should be mentioned and discussed.

8. Power analysis findings should be provided to justify the sample size.

9. Details of statistical analysis should be provided.

10. In discussion it is not possible to say that findings "support structural assessment reforms"as the policy leap not justified by cross-sectional, observational data. Rewording is needed.

11. Discussion states that "simply 'having strategies' is not enough" but earlier emphasizes that 26% had "no specific strategy" as a vulnerable group. If strategies don't affect ranking, why is this vulnerability meaningful? Clarify.

**Reviewer**	**2**
**Name**	**Eley, Diann**
**Affiliation**	**University of Queensland, Faculty of Medicine**
**Date**	**21-Aug-2026**
**COI**	**None**

---

1. Is the research question or study objective clearly defined?

• Yes, but the research question and justification for could have been more detailed. The research question and objective should coincide with the study conclusions. In fact, the Conclusion summarises the study objectives well.

Also - The introduction could provide more background or justification for using the transactional stress model, and coping reservoir frameworks. These are not well described in the introduction which is where I would expect them to be. It is mentioned that they use these to interpret different profiles of anxiety, but why and what are these profiles of

anxiety. There is some literature around this, but I don't feel they have described how it is relevant to their study and the transactional stress model, and coping reservoir frameworks.

4. Are the methods described sufficiently to allow the study to be repeated?

• There are some details missing in the methods. Was the completion of the survey monitored?

• There are several acronyms that have not been spelled out. I realise that some may refer to a survey or condition, but others are confusing, and I cannot find their meaning. For example, (line 35, page 8) (e.g., "Stress ECOS," is listed along with medical hypnosis, narrative medicine). Is Stress ECOS a separate module or activity or does it stand for these others?

7. If statistics are used, are they appropriate and described fully?

• Because the statistics are largely descriptive, I wonder I they considered looking at effect size i.e., Cohen's d?

9. Do the results address the research question or objective? yes

10. Are they presented clearly?

• Figures 1, 2 and 3 are not familiar to me and do not fine them particularly helpful. That is a limitation of mine but a one sentence explanation of the message takeaway from each figure would be helpful to many readers.

11. Are the discussion and conclusions justified by the results

• I'm still not sure what a transactional stress model or lens means in the context of the discussion. It has not (in my opinion) been described yet.

• Page 13, line 35-58 – This paragraph is confusing. It starts out, "We first noticed that stress….." What is the context here? It suggests that that stress scores have already been measured before, but are they in this cohort or is this just from the literature? Perhaps an opening sentence recapping what this study looked and why would be helpful.

• The discussion very long. The conclusions do not present anything new but reinforces strategies to assist schools and students in addressing anxiety.

13. Is the supplementary reporting complete (e.g. trial registration; funding details; CONSORT, STROBE or PRISMA checklist)?

• Yes STROBE

VERSION 1 - AUTHOR RESPONSE

Reviewer: 1
Dr. Lut Tamam, Çukurova University Medical School
Comments to the Author:
The current study examines stress, coping behaviors, and anxiety among 192 French

final-year medical students (50% response rate) preparing for a high-stakes national ranking examination (EN). The study found pervasive stress affecting 92% of students, with significant impacts on attention (83%), sleep (79%), and learning capacity (64%). While 86% engaged in regular sports and 61% practiced breathing exercises, 32% had consulted a psychologist/psychiatrist and 15% used benzodiazepines. Trait anxiety levels did not significantly correlate with final national ranking, and self-reported coping strategies showed no association with better performance. The study is an original one within the scope of the journal. My comments are as follows;

1. The subtitle "a transactional stress perspective" suggests inclusion of theoretical depth in the study which is not the case. It should be removed or revised.
We agree that the subtitle could over-promise theoretical depth. In line with the editor's request to include the study design, we have removed "a transactional stress perspective" and clarified the design in the title that is now "Stress, coping profiles and anxiety among French final-year medical students in a high-stakes national ranking examination: a cross-sectional study".

2. Lazarus & Folkman's transactional theory and the "coping reservoir" model are mentioned briefly but not operationalized. They should be integrated into testable hypotheses with clear variables.
3. Hypothesis should be more clear and straightforward with proposed direction of associations.
We appreciate this and have clarified how these models informed specific, testable hypotheses. We now explicitly state the variables and expected directions (e.g. active coping behaviours, lower anxiety, less medicalised coping, and patterns of ranking). We changed the last paragraph of the introduction to: "Guided by Lazarus and Folkman's transactional theory and the coping reservoir model, we formulated three exploratory hypotheses: (1) students engaging in structured, active coping behaviours (regular physical activity, breathing exercises) would report lower trait anxiety and less reliance on medicalised coping (benzodiazepines and other psychotropics) than those without such behaviours; (2) higher levels of trait anxiety would be associated with greater functional impact of stress and more frequent psychological follow-up, but only modestly with final exam ranking; and (3) distinct stress–coping profiles (combinations of anxiety, coping behaviours and medicalised coping) would emerge across the ranking spectrum, illustrating different ways students maintain performance within a performance-oriented system."

4. The novelty of the study and the gap it fills out should also be mentioned.
We have now explicitly stated the novelty and gap: this is, to our knowledge, the first study to examine stress, coping and anxiety in the context of the newly reformed French national ranking examination (EN combining EDN and nOSCE) using a transactional/coping reservoir lens. We added at the beginning of the last paragraphe of the introduction section the following: "To our knowledge, no prior study has examined how French final-year medical students experience and manage stress in the context of the newly reformed EN that combines EDN and nOSCE, nor how stress-coping profiles relate to national ranking when interpreted through contemporary stress and wellbeing models. This study therefore addresses a gap between descriptive reports of exam anxiety and conceptual models of stress and coping in health professions education."

5. The scales are administered "immediately post-nOSCE," the STAI-Y state scores reflect post-exam relief or exhaustion as much as during-exam anxiety. This might compromise validity. It should be mentioned in limitations in discussion.

We agree that administering STAI-Y-state immediately post-nOSCE may capture a mix of exam-related anxiety, relief and exhaustion. However, we chose to do this to have the maximum response rate as possible. We have added this explicitly as a limitation in the Discussion page 17: "Furthermore, STAI-Y state scores were collected immediately after completion of the nOSCE; they may therefore reflect a composite of anticipatory anxiety, perceived performance, relief and fatigue, which could dilute the specificity of "during-exam" stress and should be interpreted accordingly."

6. The 40-item instrument used in the study was "developed by a multidisciplinary team" and piloted with only 5 students + 2 faculty. This is insufficient for establishing psychometric properties. Reliability metrics (Cronbach's alpha) should be reported for this instrument.

We agree that the preliminary piloting is insufficient for full psychometric validation. The 40-item questionnaire was designed to capture context-specific information rather than to function as a new validated scale. We did not perform a formal reliability analysis (Cronbach's alpha) for this instrument, and we now explicitly acknowledge this as a methodological limitation, while relying on STAI-Y for psychometrically robust anxiety measurement. We added the following sentences:

- Page 7: "The instrument was internally piloted with five senior medical students and two faculty members to confirm face validity, logical flow and comprehensibility, but no formal reliability indices (e.g. Cronbach's alpha) were computed; thus, the 40-item questionnaire should be considered a structured data-collection tool rather than a fully validated psychometric scale, and this is acknowledged as a limitation."
- In the discussion section page 16: "In addition, while the bespoke 40-item questionnaire underwent face-validity piloting, we did not conduct a full psychometric validation including internal consistency analyses, so findings based on these items should be interpreted with this limitation in mind and complemented by the more robust STAI-Y measures."

7. Absence of data on prior academic performance, socioeconomic status, or baseline mental health history that could be crucial for explaining both anxiety and ranking, is an important limitation that should be mentioned and discussed.

We agree. We did not collect detailed data on prior academic performance, socioeconomic status or baseline mental health, and we now highlight this as a constraint on explanatory interpretation and potential confounding. We added the following sentence in the discussion section page 17: "We did not collect detailed information on prior academic performance, socioeconomic status or baseline mental health (e.g. pre-existing anxiety or depressive disorders), which limits our ability to disentangle the relative contributions of these factors to both anxiety and ranking and introduces potential residual confounding."

8. Power analysis findings should be provided to justify the sample size.

This study was designed as an exploratory, whole-cohort survey of all students sitting the nOSCE at a single faculty, rather than a hypothesis-driven trial with an a priori sample size calculation. We therefore did not perform a formal power analysis; we have now indicated this explicitly in the Methods page 7: "As this was an exploratory survey of all eligible students at the faculty, no formal a priori power calculation was performed; instead, the sample size was determined by the size of the final-year cohort present at the nOSCE."

9. Details of statistical analysis should be provided.

We have expanded the Statistical analysis section to specify which tests were used for which comparisons, and how assumptions were handled.

10. In discussion it is not possible to say that findings "support structural assessment reforms" as the policy leap not justified by cross-sectional, observational data. Rewording is needed.
We have softened the wording to "suggest" and "highlight" the need for reflection on assessment structures, explicitly framing our implications as conceptual and hypothesis-generating rather than prescriptive:

- In the abstract: "these results highlight the need for reflection on structural assessment practices and for longitudinal institutional strategies to protect learner wellbeing.
- In the concluding paragraph: "Our findings, coupled with the observed 15% rate of benzodiazepine use and 32% rate of professional psychological consultation, suggest that high-stakes ranking assessments warrant critical reflection from the perspective of learner wellbeing and coping capacity, and they provide empirical grounds for institutions to consider structural and curricular approaches that may mitigate documented stress and functional impairment, while recognising that causal and policy inferences require further longitudinal and interventional research"

11. Discussion states that "simply 'having strategies' is not enough" but earlier emphasizes that 26% had "no specific strategy" as a vulnerable group. If strategies don't affect ranking, why is this vulnerability meaningful? Clarify.
Thank you for highlighting this potential inconsistency. Our intention was to distinguish performance outcomes from wellbeing outcomes. While self-reported strategies were not associated with ranking, students without any strategies (and often without regular physical activity) may still be vulnerable in terms of wellbeing, burnout risk or reliance on medicalised coping, within the coping reservoir framework. We have clarified that the vulnerability refers to mental health and resilience, rather than exam ranking, in the discussion page 15: "simply 'having strategies' is not sufficient to guarantee a ranking advantage—quality, timing, personal fit and context likely determine their effectiveness for both performance and wellbeing. Nonetheless, within a coping reservoir perspective, students reporting no specific strategies and minimal physical activity may represent a subgroup at greater risk of emotional exhaustion, avoidance coping or increased reliance on pharmacological strategies, even if this did not manifest as poorer ranking in our cross-sectional data."

Reviewer: 2
Dr. Diann Eley, University of Queensland
Comments to the Author:
1. Is the research question or study objective clearly defined?
• Yes, but the research question and justification for could have been more detailed. The research question and objective should coincide with the study conclusions. In fact, the Conclusion summarises the study objectives well.
We have added the detailed objectives at the end of the introduction section as also requested by reviewer #1: "Guided by Lazarus and Folkman's transactional theory and the coping reservoir model, we formulated three exploratory hypotheses: (1) students engaging in structured, active coping behaviours (regular physical activity, breathing exercises) would report lower trait anxiety and less reliance on medicalised coping (benzodiazepines and other psychotropics) than those without such behaviours; (2) higher levels of trait anxiety would be associated with greater functional impact of stress and more frequent psychological

follow-up, but only modestly with final exam ranking; and (3) distinct stress–coping profiles (combinations of anxiety, coping behaviours and medicalised coping) would emerge across the ranking spectrum, illustrating different ways students maintain performance within a performance-oriented system."

At the same time, reviewer #1 also requested how the novelty gap was bridged, we added this to the introduction section: "To our knowledge, no prior study has examined how French final-year medical students experience and manage stress in the context of the newly reformed EN that combines EDN and nOSCE, nor how stress-coping profiles relate to national ranking when interpreted through contemporary stress and wellbeing models. This study therefore addresses a gap between descriptive reports of exam anxiety and conceptual models of stress and coping in health professions education"

Also - The introduction could provide more background or justification for using the transactional stress model, and coping reservoir frameworks. These are not well described in the introduction which is where I would expect them to be. It is mentioned that they use these to interpret different profiles of anxiety, but why and what are these profiles of anxiety. There is some literature around this, but I don't feel they have described how it is relevant to their study and the transactional stress model, and coping reservoir frameworks.

We thank the reviewer for this helpful suggestion. We have expanded the Introduction to more clearly justify the use of Lazarus & Folkman's transactional theory and the coping reservoir model, explaining why these frameworks are relevant to high-stakes examinations, how they conceptualise "profiles" of stress and coping, and how our variables operationalise these constructs. We also clarify that by "profiles of anxiety" we refer to combinations of trait/state anxiety, coping behaviours and medicalised coping that map onto different appraisal and coping configurations (e.g. high anxiety/high performance vs low anxiety/low performance). We added the following sentences page 6: "In this literature, different "profiles" of distress and coping arise depending on how demands are appraised (e.g. threat vs challenge), what resources are available, and whether coping is behavioural, cognitive, social or medicalised [17,18]. In our study, profiles of anxiety and coping are operationalised as combinations of *State-Trait Anxiety Inventory-Y*, (STAI-Y) state and trait scores, self-reported coping behaviours (sport, breathing exercises, other strategies) and use of professional and pharmacological coping, examined in relation to national ranking within a performance-oriented system." We aimed at staying quite concise but this section can also be further expanded if you request it.

4. Are the methods described sufficiently to allow the study to be repeated?

• There are some details missing in the methods. Was the completion of the survey monitored?

• There are several acronyms that have not been spelled out. I realise that some may refer to a survey or condition, but others are confusing, and I cannot find their meaning. For example, (line 35, page 8) (e.g., "Stress ECOS," is listed along with medical hypnosis, narrative medicine). Is Stress ECOS a separate module or activity or does it stand for these others?

We appreciate these requests for clarity. We have now specified that the paper questionnaire was completed in the exit room, unsupervised except for the presence of staff to distribute and collect forms, and that students placed completed questionnaires in a sealed box, with no time limit other than the closure of the room. We added the following sentence page 7: "Completion was not individually monitored or timed; students completed the survey

independently in the exit room, and staff were present only to distribute and collect forms and to close the room at the end of the session."

We have also spelled out "Stress ECOS" as a specific online faculty-led stress-management programme targeting OSCE ("ECOS" in French) preparation, distinct from medical hypnosis and narrative medicine modules. We modified the text page 8: "Participation in elective university-led wellness modules was recorded, including an online faculty programme specifically focused on stress reduction for OSCE preparation ("Stress ECOS"), as well as separate options in medical hypnosis and narrative medicine. Personal coping strategies (e.g. breathing exercises, yoga, meditation) were also recorded."

7. If statistics are used, are they appropriate and described fully?

• Because the statistics are largely descriptive, I wonder I they considered looking at effect size i.e., Cohen's d?

We thank the reviewer for this suggestion. Given the exploratory, descriptive nature of the study and the focus on broad patterns rather than precise effect estimation, we did not originally calculate effect sizes. We agree that effect sizes would add further quantitative nuance, and we now acknowledge this omission as a limitation and state that future work should incorporate effect-size reporting (e.g. Cohen's d or odds ratios) for key comparisons. As also highlighted by reviewer #1, we have now indicated this explicitly in the Methods page 7: "As this was an exploratory survey of all eligible students at the faculty, no formal a priori power calculation was performed; instead, the sample size was determined by the size of the final-year cohort present at the nOSCE."

9. Do the results address the research question or objective? yes

10. Are they presented clearly?

• Figures 1, 2 and 3 are not familiar to me and do not fine them particularly helpful. That is a limitation of mine but a one sentence explanation of the message takeaway from each figure would be helpful to many readers.

We agree that clear "take-home" messages for each figure will aid readers. We have added a brief explanatory sentence to each figure legend summarising the key message:

- Fig 1: nOSCE-related stress was rated slightly higher than EDN-related stress on a 0–10 scale, with most students reporting high stress for both components.
- Fig 2: STAI-Y state and trait scores were generally in the low-to-moderate range, with women showing higher median trait anxiety than men and only a moderate correlation between state and trait anxiety.
- Fig 3: Across the ranking spectrum, many students with low–moderate trait anxiety achieved good rankings, and extreme anxiety profiles (very low vs very high) were distributed across both high and low ranks, illustrating weak linkage between trait anxiety and performance.

11. Are the discussion and conclusions justified by the results

• I'm still not sure what a transactional stress model or lens means in the context of the discussion. It has not (in my opinion) been described yet.

• Page 13, line 35-58 – This paragraph is confusing. It starts out, "We first noticed that stress….." What is the context here? It suggests that that stress scores have already been measured before, but are they in this cohort or is this just from the literature? Perhaps an opening sentence recapping what this study looked and why would be helpful.

We appreciate this feedback. We have (a) added a short opening sentence at the start of the Discussion to recapitulate the study aim, (b) inserted a brief paragraph explicitly explaining what we mean by a "transactional lens" in relation to our findings, and (c) simplified the opening "We first noticed…" paragraph to make clear that it refers to our own results rather than prior literature. We have also lightly streamlined the Discussion by removing some repetitive sentences

- At the beginning of the discussion page 13 we added: This study examined stress, coping behaviours, anxiety and national ranking among French final-year medical students sitting a newly implemented high-stakes national examination (EN), and interpreted the observed profiles of distress and coping through transactional and coping reservoir frameworks.
- Followed by: "Within a transactional stress lens, our findings can be understood as patterns of appraisal and coping: students face high external demands (the EN), appraise these demands as threat or challenge, draw on available resources (sport, breathing exercises, psychological support), and adopt coping responses that are behavioural, cognitive, social or medicalised. The coping reservoir model further suggests that repeated exposure to such demands and coping choices either depletes or replenishes students' resilience over time, which helps explain how some students maintain performance despite high anxiety and extensive medicalised coping, while others maintain wellbeing with more moderate performance."
- And followed by, at the beginning of the second paragraph of the discussion: "In our cohort, stress was pervasive and intense: students reported high numerical stress scores for both exam components, especially the nOSCE, and a broad impact on attention, sleep, learning and eating, with additional repercussions on social life, mood and somatic health"

• The discussion very long. The conclusions do not present anything new but reinforces strategies to assist schools and students in addressing anxiety.
We acknowledge that the Discussion is relatively detailed. We have streamlined some repetitive segments and emphasised that our main conceptual contribution is to illustrate, in a new national examination context, that high performance can coexist with very different stress-coping profiles and that coping behaviours appear more tightly linked to wellbeing than to ranking. We also explicitly frame our recommendations as hypothesis-generating rather than definitive policy prescriptions. Finally, we added the following sentence in the conclusion: "Conceptually, the results highlight that high-stakes ranking examinations can produce diverse stress–coping configurations, with similar ranking outcomes arising from very different combinations of anxiety, behavioural coping and medicalised coping, which supports the use of transactional and coping reservoir models to understand learner wellbeing in performance-oriented systems"

13. Is the supplementary reporting complete (e.g. trial registration; funding details; CONSORT, STROBE or PRISMA checklist)?
• Yes STROBE
