# Supplementary material for: Stress, coping profiles and anxiety among French final-year medical students in a high-stakes national ranking examination: a cross-sectional study
Source: BMJ Open. 2026 Sep 8;16(9):e125171. doi: 10.1136/bmjopen-2026-125171 (PMC13560983; doi:10.1136/bmjopen-2026-125171)
Supplement: Supplementary file 2 [file bmjopen-16-9-s001.docx]

**Dear students,**

**We would like to evaluate the stress management of medical students during national OSCEs. We guarantee this questionnaire to be fully anonymous: your student number will only be used to consider your success rate in the exam and to match you anonymously with students who have undertaken specific stress management training. Thank you all!**

**GENERAL PRESENTATION**

1. **Are you?**A woman A man Don't say anything
2. **Your year of birth? _____**
3. **Your student number** *(to match the final result)?* **_____________________**
4. **Do you exercise regularly?**

- No
- Walking and/or jogging
- Football, rugby, handball... (Team Sports)
- Martial Arts
- Yoga, pilate
- Other: ____________________________

5. **Do you have a regular professional activity in parallel with your 6th year?**

- Yes No

6. **Have you already taken any of the following courses?**

- Optional module "Medicine, Meditation and Storytelling"
- Optional module in DFASM2 "Awareness of medical hypnosis"
- "StressECOS" training
- Other training courses on stress management: ____________

7. **Among those who registered for the "StressECOS" training, were you able to attend:**

- All 5 sessions
- At least 3 sessions
- Less than 3 sessions
- No sessions

## I. **ASSESSMENT OF STRESS IN GENERAL**

8. **During your medical studies, what means(s) of stress management did you implement?**

- None
- Breathing exercises (cardiac coherence, abdominal breathing, etc.)
- Mindfulness Meditation
- Auto-hypnosis
- Yoga
- Other: ____________________________

9. **In the past year, have you ever used a benzodiazepine for anxiolytic purposes?**

- Yes No

10. **Over the past year, do you think your stress has been able to:**

- Disrupting your ability to learn
- Disrupting your attentional abilities
- Impact on the quality of your sleep
- Affect your meal intake
- Other: ____________________________
- No Element Cited

11. **During the past year, have you been followed by a psychologist or psychiatrist for the indication "psychological stress management support"?**

- Yes No

## II. **DURING THE TESTS**

12. **From 0 to 10, how do you rate your stress level during nOCSE? =** ________
13. **From 0 to 10, how do you rate your stress level during EN? =** ________
14. **In the hours leading up to the OSCE, what means(s) of stress management did you implement?**

- None
- Breathing exercises (cardiac coherence, abdominal breathing, etc.)
- Mindfulness Meditation
- Auto-hypnosis
- Yoga
- Other: ____________________________

15. **Between 2 OSCE stations, what means of stress management have you implemented?**

- None
- Breathing exercises (cardiac coherence, abdominal breathing, etc.)
- Mindfulness Meditation
- Auto-hypnosis
- Other: ____________________________

16. **Between the 1st and 2nd day, what means(s) of stress management did you implement?**

- None
- Breathing exercises (cardiac coherence, abdominal breathing, etc.)
- Mindfulness Meditation
- Auto-hypnosis
- Yoga
- Other: ____________________________

17. **How would you describe your sleep this week?**

- Very bad
- Bad
- Medium
- Good
- Very good

**IV. CURRENTLY, how do you feel? *(STAI-Y1 scale state)***

***For each proposal, tick the box that best corresponds to your condition:***

|  | Not at all | Somewhat | Moderately so | Very much so |
| --- | --- | --- | --- | --- |
| 1. I feel calm |  |  |  |  |
| 2. I feel safe |  |  |  |  |
| 3. I'm tense |  |  |  |  |
| 4. I feel strained |  |  |  |  |
| 5. I feel at ease |  |  |  |  |
| 6. I feel moved, upset |  |  |  |  |
| 7. I am presently worrying over possible misfortunes |  |  |  |  |
| 8. I feel satisfied |  |  |  |  |
| 9. I feel frightened |  |  |  |  |
| 10. I feel good, comfortable |  |  |  |  |
| 11. I feel confident |  |  |  |  |
| 12. I feel nervous |  |  |  |  |
| 13. I feel jittery |  |  |  |  |
| 14. I feel indecisive |  |  |  |  |
| 15. I'm relaxed |  |  |  |  |
| 16. I feel content |  |  |  |  |
| 17. I'm worried |  |  |  |  |
| 18. I feel confused |  |  |  |  |
| 19. I feel steady |  |  |  |  |
| 20. I feel pleasant |  |  |  |  |

**V. IN GENERAL, how do you feel in your daily life? *(STAI-Y2 scale line)***

***For each proposal, tick the box that best corresponds to your condition:***

|  | Almost never | Sometimes | Often | Almost always |
| --- | --- | --- | --- | --- |
| 21. I feel pleasant |  |  |  |  |
| 22. I feel nervous and restless |  |  |  |  |
| 23. I feel satisfied with myself |  |  |  |  |
| 24. I wish I could be as happy as others seem to be |  |  |  |  |
| 25. I feel like a failure |  |  |  |  |
| 26. I feel rested |  |  |  |  |
| 27. I am "calm, cool, and collected" |  |  |  |  |
| 28. I feel that difficulties are piling up so that I cannot overcome them |  |  |  |  |
| 29. I worry too much over something that really doesn't matter |  |  |  |  |
| 30. I'm happy |  |  |  |  |
| 31. I have disturbing thoughts |  |  |  |  |
| 32. I lack self-confidence |  |  |  |  |
| 33. I feel secure |  |  |  |  |
| 34. I make decisions easily |  |  |  |  |
| 35. I feel inadequate |  |  |  |  |
| 36. I am content |  |  |  |  |
| 37. Some unimportant thought runs through my mind and bothers me |  |  |  |  |
| 38. I take disappointments so keenly that I can't put them out of my mind |  |  |  |  |
| 39. I'm a steady person |  |  |  |  |
| 40. I get in a state of tension or turmoil as I think over my recent concerns and interests |  |  |  |  |

**VI.	FREE COMMENTS**

Do not hesitate to leave your impressions/remarks/proposals, concerning this questionnaire and/or stress management in the context of exams, it will be VERY much appreciated! 🙂
